# Aortic valve tear with severe aortic regurgitation following blunt chest trauma

**DOI:** 10.1186/1749-8090-6-84

**Published:** 2011-06-19

**Authors:** Weidong Li, Yiming Ni, Xin chen, Liang Ma

**Affiliations:** 1 Department of Cardiothoracic Surgery, 1st Affiliated Hospital, School of Medicine, Zhejiang University, Hang Zhou, 310003, China

## Abstract

An aortic valve tear associated with aortic regurgitation following blunt chest trauma is seldom seen. In this case, a 55-year-old man sustained a non-penetrating chest injury caused by a sudden fall from 10 meters. This led to a sizable tear in the left coronary cusp associated with severe aortic insufficiency. The case was treated successfully by surgical replacement of the aortic valve with a mechanical prosthesis.

## Background

Aortic valve tear is a rare complication of blunt chest trauma which can lead to severe aortic regurgitation. Blunt chest traumas are frequently caused by traffic accidents or falls from a great height. Here, we present a case of a tear in the aortic valve associated with severe aortic regurgitation following blunt chest trauma caused by falling from a great height.

## Case report

A 55-year-old man suffered from a sudden fall from 10 meters. He was immediately sent to a local hospital and was diagnosed with multiple bone fractures including pelvis, right acetabulum and right radius. He also complained of shortness of breath and transthoracic echocardiography (TTE) showed aortic valve prolapse associated with moderate aortic regurgitation. Due to the stable hemodynamic situation, conservative treatment of the bone fractures was given first.

Four months later, he was transferred to our hospital for further treatment of aortic prolapse. On physical examination, the patient showed no acute distress. His blood pressure was 135/65 mmHg and pulse rate was 81 beats per min. A grade 4/6 diastolic murmur and significant thrill was heard in the aortic area. An electrocardiogram showed no abnormality. Chest X-ray did not reveal pulmonary congestion and pulmonary inflammation. TTE revealed severe aortic regurgitation and the prolapse of the left coronary cusp. These investigations confirmed massive aortic regurgitation, which was thought to be due to the rupture of the left coronary cusp.

The patient underwent surgery. Following aortic cross-clamping and cardioplegic arrest, the ascending aorta was opened on total cardiopulmonary bypass. We found a tear of about 10 mm in the left coronary cusp which caused the prolapse of this cusp. No abnormalities were found in the aortic wall and aortic valve annulus. The aortic valve was removed and replaced with a No. 21 Carbomedics mechanical prosthesis.

The post-operative period was uneventful and the patient was discharged 10 days after operation. The structure and function of the prosthetic aortic valve was good as assessed by ultrasound cardiogram (UCG) during the follow-up period and no aortic valve regurgitation was found. The patient remains well more than 1 year after operation.

## Discussion

Blunt chest trauma often occurs after high-speed traffic accidents or after falls. The common injury is myocardial contusion which presents with transitory shortness of breath, increasing cardiac enzymes and mild abnormality of the electrocardiogram. The patient usually recovers with conservational treatment.

But an aortic valve tear following blunt chest trauma is very rare. Since Penderleath found the first case of acute aortic valve tear in the biopsy of one traffic accident victim in 1830 [1], no more than 100 cases were reported all over the world up to 2002 [2].

The mechanism causing aortic valve lesions is considered to be a sudden increase in intracardiac pressure during a vulnerable phase of the cardiac cycle, especially during early diastole, when the transaortic gradient is maximal [3]. This leads to a tear or rupture of the aortic cusp. Usually only one is damaged, and the non-coronary cusp is most commonly involved. In the reports between 1994 and 2005, 28 aortic cusp lesions were decribed. Fourteen were non-coronary, 10 right coronary and only 4 left coronary [4]. The possible mechanism is that the outflow into the right and left coronary arteries may decrease the hemodynamic stress to the left and right aortic valve cusps. Since the right coronary cusp is closer to the anterior chest wall, it may suffer higher pressure and be more likely to tear than the left cusp. In the case presented here, the left coronary cusp was torn, a very rare occurrence.

The diagnosis of traumatic aortic valve tear is not difficult: acute or progressive heart failure, a new heart murmur and a history of blunt chest trauma. But it often takes more than one week from the trauma to the onset of heart failure. Furthermore, the existence of multiple injuries may cover the chest symptoms. So more attention should be paid to patients with blunt chest trauma in clinical experience. UCG is very useful in defining injury of the aortic valve and confirming the presence of aortic regurgitation.

The decision to operate is very important and can be decided on the basis of heart function and the systemic situation. If the aortic regurgitation does not impair hemodynamic stability, or if there are other lesions that may increase operative risk, as in our case, delayed instead of emergency operation is advised, as this allows the surgeon to select the best conditions for intervention. Since there may be an asymptomatic period for a week or more, surgeons treating these patients should be aware of the potential for rapid decompensation and the need for an emergency operation.

The indications for the valve replacement or repair depend on many factors: the extent of injury to the cusp, the number of cusps or commissures involved, and the ability of the surgeon to performance aortic valve repair. Prosthetic valve replacement is preferred in cases with a complicated tear or multiple lesions of the cusp [4]. However, in some cases where the aortic root or valve commissures are involved, replacement with a composite mechanical valved-dacron graft conduit should be considered [5]. Repair methods such as direct suture or auto-pericardial patch are suitable for a simple and regular rupture in one cusp [6]. But one type of rupture of the cusp should receive special attention. One report showed a visible tear of the non-coronary cusp during operation without any lesions of the left and right coronary cusps. While microscopic examination of the pathological tissue also revealed a tear in the left cusp, which had suffered no laceration [7]. This finding seems to indicate that it may not be possible to diagnose the extent of a rupture by direct visual observation during surgery. In this case, repairing only the non-coronary cusp may lead to an insufficiency of the left coronary cusp in the future. So we think that even in the lesion of a single valve cusp, if the rupture is severe, the potential rupture of other cusps should be considered and a prosthetic valve replacement is a good and safe choice.

## Consent

Written informed consent was obtained from the patient for publication of this case report and accompanying images. A copy of the written consent is available for review by the Editor-in-Chief of this journal.

## Competing interests

The authors declare that they have no competing interests.

## Authors' contributions

WL wrote the draft of the manuscript and obtained the written consent. XC performed the literature review and participated in the manuscript writing. NM was the cardiology consultant. LM helped to the final writing of the paper and gave final approval of the manuscript. All authors have read and approved the final manuscript.

**Figure 1:**
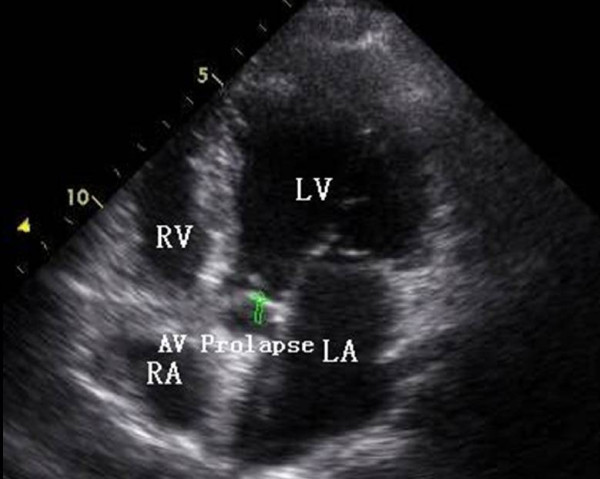
**TTE from apical 5-chamber plane view showing prolapse of the left coronary cusp (green arrow)**. AV: aortic valve; LA: left atrium; LV: left ventricle; RA: right atrium; RV: right ventricle.

**Figure 2:**
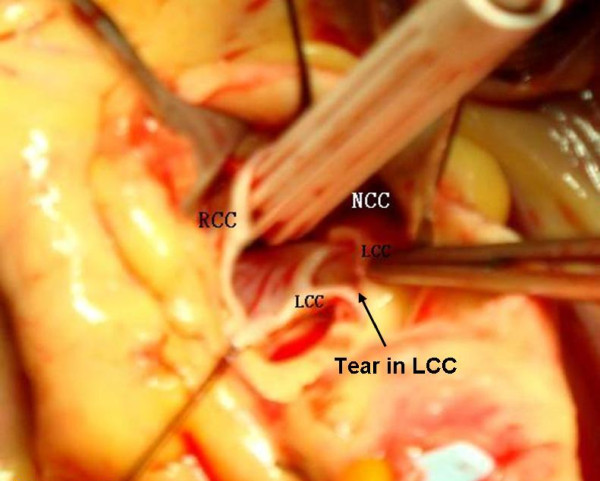
**A 10-mm tear in the left coronary cusp**. LCC: left coronary cusp; RCC: right coronary cusp; NCC: non coronary cusp.
